# Enabling ambulatory movement in wearable magnetoencephalography with matrix coil active magnetic shielding

**DOI:** 10.1016/j.neuroimage.2023.120157

**Published:** 2023-05-05

**Authors:** Niall Holmes, Molly Rea, Ryan M. Hill, James Leggett, Lucy J. Edwards, Peter J. Hobson, Elena Boto, Tim M. Tierney, Lukas Rier, Gonzalo Reina Rivero, Vishal Shah, James Osborne, T. Mark Fromhold, Paul Glover, Matthew J. Brookes, Richard Bowtell

**Affiliations:** a Sir Peter Mansfield Imaging Centre, School of Physics and Astronomy, University of Nottingham, University Park, Nottingham NG7 2RD, UK; b Cerca Magnetics Limited, Unit 2 Castlebridge Office Village, Kirtley Drive, Nottingham NG7 1LD, UK; c School of Physics and Astronomy, University of Nottingham, University Park, Nottingham NG7 2RD, UK; d Wellcome Centre for Human Neuroimaging, UCL Queen Square Institute of Neurology, University College London, London WC1N 3AR, UK; e QuSpin Inc., 331 South 104th Street, Suite 130, Louisville, CO 80027, USA

## Abstract

The ability to collect high-quality neuroimaging data during ambulatory participant movement would enable a wealth of neuroscientific paradigms. Wearable magnetoencephalography (MEG) based on optically pumped magnetometers (OPMs) has the potential to allow participant movement during a scan. However, the strict zero magnetic field requirement of OPMs means that systems must be operated inside a magnetically shielded room (MSR) and also require active shielding using electromagnetic coils to cancel residual fields and field changes (due to external sources and sensor movements) that would otherwise prevent accurate neuronal source reconstructions. Existing active shielding systems only compensate fields over small, fixed regions and do not allow ambulatory movement. Here we describe the matrix coil, a new type of active shielding system for OPM-MEG which is formed from 48 square unit coils arranged on two planes which can compensate magnetic fields in regions that can be flexibly placed between the planes. Through the integration of optical tracking with OPM data acquisition, field changes induced by participant movement are cancelled with low latency (25 ms). High-quality MEG source data were collected despite the presence of large (65 cm translations and 270°rotations) ambulatory participant movements.

## 1. Introduction

Characterization of the neural correlates of walking, gait and balance in conditions such as Parkinson’s disease (Morris et al., 2001), stroke (Den Otter et al., 2005) and concussion (Kevin et al., 2001) could be used to provide significant insight into diagnosis, stratification and prognosis. Indeed, access to non-invasive assessment of brain function during ambulatory movement would open up a wealth of interesting neuroscientific paradigms. However, in this context the available functional neuroimaging technology places significant limitations on experimental design and data quality. For example, functional MRI (fMRI) requires participants to be enclosed in the scanner bore and remain motionless. Whilst electroencephalography (EEG) has excellent temporal resolution (~1 ms) and forms a wearable technology, enabling ambulatory movement studies using treadmills ramps and stairs (Brantley et al., 2018; Wagner et al., 2019), it suffers from poor spatial resolution (~2 cm) (Baillet, 2017) and a high sensitivity to muscle artefacts during movement (Boto et al., 2019; Muthukumaraswamy, 2013). Functional near infrared spectroscopy (fNIRS) is similarly deployable, but (like fMRI) provides measurements of haemodynamic changes that are indirect reflections of brain function and which have relatively poor spatial and temporal resolution. A robust, accurate, electrophysiological, measure of brain function during ambulatory movement would be a powerful platform for neuroscientific studies.

Magnetoencephalography (MEG) (Cohen, 1968) measures the tiny magnetic fields generated outside the scalp by electrical activity in the brain. These measurements can be used to map the distribution of underlying neural currents with a unique combination of good spatial (~5 mm) (Barratt et al., 2018; Troebinger et al., 2014) and temporal (~1 ms) resolution (Baillet, 2017; Hämäläinen et al., 1993). MEG is a powerful neuroscientific tool, which also has clinical applications, particularly in epilepsy (Rampp et al., 2019). However, implementation of MEG presents significant challenges. Conventional MEG systems use fixed arrays of superconducting magnetic field sensors placed inside a helmet within a cryogenic dewar. The fixed helmet size limits the range of participant movement that can be tolerated during a scan. Recent years have seen the development of ‘wearable’ MEG systems based on arrays of optically pumped magnetometers (OPMs) (see(Brookes et al. 2022) for a review). OPMs are cryogen-free sensors which allow the design of sensor arrays that can be adapted to the application of interest, and crucially can move with the participant (Boto et al., 2018; Hill et al., 2020).

OPMs with the sensitivity $\left(\sim 10-20fT/\sqrt{}Hz\right)$ needed to detect MEG signals (such as those commercially available from QuSpin Inc. (Louisville, Colorado, USA) and FieldLine Inc. (Boulder, Colorado, USA)) (see (Tierney et al. 2019) for a review of OPM physics), are operated around a narrow, zero-field resonance with a dynamic range of just a few nanotesla (nT); meaning data quality is very sensitive to small changes in magnetic field such as those generated by nearby temporally varying sources and by changes in position and orientation of the sensor in a non-zero background field. As the magnetic field experienced by the sensor moves further from zero, the OPM response function becomes increasingly non-linear, eventually producing complete signal saturation if the field is too high. This change in the sensor gain can adversely affect the quality of source modeling, both in terms of the fidelity of reconstructed time-courses of neural activity and the localization of sources within the brain (Borna et al., 2022; Nugent et al., 2022).

OPMs are generally operated within a magnetic shield, which is typically a magnetically shielded room (MSR) or cylinder (MSC). Multiple layers of material with a high magnetic permeability (usually MuMetal, a nickel-iron alloy) are used to screen low-frequency magnetic fields (e.g. those generated by passing vehicles or elevators) in combination with a layer of a material with a high electrical conductivity (e.g. copper/aluminium) to screen higher frequency fields (e.g. those generated by mains electricity) (Hoburg, 1995). Although high shielding factors can be achieved, often a small magnetic field (between 10 and 50 nT) remains inside the shield due to residual magnetization of the high-permeability shielding material. Demagnetization coils can reduce the remnant field to 2–5 nT (Altarev et al., 2015; Voigt et al., 2013). Low-frequency (typically <5 Hz) magnetic field changes induced by sources such as passing vehicles, elevators, etc. are not shielded as strongly by large passive shields as higher frequency interference sources. Different environments have reported various levels of field changes such as ±1 nT over 10 min (Hill et al., 2020; Iivanainen et al., 2019) or even as much as ±5 nT in just a few seconds (Hill et al., 2022; Holmes et al., 2019). The size and temporal form of such ‘drifts’ largely depend on the type of source and its proximity to the shield.

To compensate the remnant magnetic field, and address field drifts, electromagnetic coils can be used to ‘actively’ shield equipment by generating a field which is equal and opposite to the field in the MSR. Typically, active shielding systems use a series of coils which each generate a known field profile (e.g. Helmholtz coils). Three coils can be used to generate the three uniform magnetic field components and a further five coils generate the linear spatial magnetic field gradients (Holmes et al., 2018; Iivanainen et al., 2019). The remnant field and its gradients are measured and, from the known field or field gradient produced per unit of coil current (termed the coil efficiency), appropriately chosen currents are applied to the coils to compensate the field. If the field is monitored via fixed reference sensors, this process can be operated in constant feedback mode to cancel low-frequency drifts (Holmes et al., 2019; Iivanainen et al., 2019).

Active shielding has been crucial to the development of OPM-MEG (Borna et al., 2020; Holmes et al., 2019, 2018; Iivanainen et al., 2019; Marhl et al., 2022; Rea et al., 2021; Zhang et al., 2020). Our previous work described a bi-planar coil system comprising two $1.6\times 1.6{\;m}^{2}$ planes separated by 1.5 m, and each containing up to eight layers of intricate windings that can be used to shield a $0.4\times 0.4\times 0.4{\;m}^{3}$ volume at the centre of the region between the two planes (Holmes et al., 2019, 2018). This system allowed seated participants to make small head movements during a scan, and enabled the acquisition of the first motion-tolerant MEG recordings with a wearable system (Boto et al., 2018). Combining bi-planar coils with precision modeling of the remnant field allowed the field to be reduced to just 290 pT in a high performance MSR (Rea et al., 2021). Other recent work has focused on improving the uniformity of field variations produced by coils operating inside magnetic shields by incorporating field distortions due to coils interacting with MuMetal (Kutschka et al., 2021; Packer et al., 2020; Zetter et al., 2020). However, although powerful, current active shielding technology does not allow measurement of brain activity during ambulatory movements.

Distinct from a single coil which is designed to generate a known field profile, multi-coil systems utilise a series of individual ‘unit coils’ with a simple geometry, e.g. circular or rectangular windings (Borna et al., 2017; Juchem et al., 2011a; Packer et al., 2022). Coil currents are calculated based on knowledge of the magnetic field pattern generated by each coil in the system, such that the vector sum of the various fields form a magnetic field with a desired strength and spatial variation. Multi-coil systems can be considered to effectively ‘re-design’ themselves, offering flexibility in application. Multi-coil systems have been used successfully in magnetic resonance imaging to shim regions that experience local field variations (Juchem et al., 2011b, 2010) as well as in the operation, localization, and calibration of OPM arrays (Borna et al., 2020; Iivanainen et al., 2022) and in the development of lightweight MSRs for OPM-MEG with a large, fixed, volume of low remnant field (Holmes et al., 2022).

Here we describe a ‘matrix coil’, which is a reconfigurable multi-coil active shielding system comprising 48 square unit coils that continually recalibrates itself to compensate the magnetic field changes experienced by a sensor array that is freely moving. By combining an array of OPMs mounted in a 3D-printed helmet with optical tracking, the efficiency of each unit coil can be rapidly evaluated for the instantaneous sensor positions. Coil currents which best compensate the measured field changes experienced by the array are calculated and applied with low latency (25 ms). The matrix coil therefore potentially forms a basis for enabling ambulatory movement in OPM-MEG recordings. In this paper, we first outline the mathematical framework used for matrix coil operation and describe the development of our system. We then detail four demonstrations of the system involving operation: (1) with a stationary array of OPMs; (2) as a seated participant makes small head movements; (3) in a phantom study to assess the effects on the system on source reconstruction accuracy and (4) during a MEG recording from an ambulatory participant.

## 2. Theory

Consider an array of N magnetic field sensors (placed in a rigid helmet such that the position and orientation of the sensors with respect to each other is fixed), each with a known position $r_{n}(x,y,z)$ and orientation $o_{n}=o_{x}\overset{ˆ}{x}+o_{y}\overset{ˆ}{y}+o_{z}\overset{ˆ}{z}$ (where $\overset{ˆ}{x},\overset{ˆ}{y}$ and $\overset{ˆ}{z}$ are the Cartesian unit vectors). The change (relative to the measurement at time t=0) in field measured by each of these sensors at time t is described by the (N rows x 1 column) vector Δb(t). The changes in field due to external sources, or movement of the array through the remnant magnetic field, that we aim to compensate will be dominated by variations which can be decomposed into low-order spherical harmonics (Seymour et al., 2021; Tierney et al., 2022, 2021) when compared with the more spatially complex underlying MEG signals. In this case, Δb(t) can be fit to a field model comprised of three uniform field terms:

$$\text{uniform gradient components}\left\{\begin{array}{ll} \alpha_{1}\overset{ˆ}{x}, & \left\{B_{x}\right\}\\ \alpha_{2}\overset{ˆ}{y}, & \left\{B_{y}\right\}\\ \alpha_{3}\overset{ˆ}{z}, & \left\{B_{z}\right\} \end{array}\right.,$$

and five magnetic field gradient components:

$$\text{field gradient components}\{\begin{array}{ll} \alpha_{4}\left(y\hat{x}+x\hat{y}\right), & \left\{\frac{dB_{x}}{dy}=\frac{dB_{y}}{dx}\right\}\\ \alpha_{5}\left(z\hat{x}+x\hat{z}\right), & \left\{\frac{dB_{x}}{dz}=\frac{dB_{z}}{dx}\right\}\\ \alpha_{6}\left(z\hat{y}+y\hat{z}\right), & \left\{\frac{dB_{y}}{dz}=\frac{dB_{z}}{dy}\right\}\\ \alpha_{7}\left(-x\hat{x}-y\hat{y}+2z\hat{z}\right), & \left\{2\frac{dB_{z}}{dz}=-\frac{dB_{x}}{dx}-\frac{dB_{y}}{dy}\right\}\\ \alpha_{8}\left(x\hat{x}-y\hat{y}\right), & \left\{\frac{dB_{x}}{dx}=\frac{dB_{y}}{dy}\right\} \end{array},$$

where the coefficient, $\alpha_{n}$, describes the strength of the $n^{\text{th}}$ component (see (Rea et al. 2021) and (Mellor et al. 2021) for further details). The equivalent magnetic field, or magnetic field gradient, for each term is shown in brackets. Using these components, the change in field can be modeled via the matrix equation

(1) A(t)α(t)=Δb(t),

where the (N rows x 8 columns) matrix A contains a model of the field or field gradient strength per unit of coefficient at each sensor. To find the coefficients captured in the (8 rows x 1 column) vector α which best maps the model to the measured fields we compute the pseudo-inverse matrix $A^{+}$ and evaluate

(2) $$\alpha =A^{+}\Delta b.$$

Now consider a series of M electromagnetic unit coils sited in the volume surrounding the sensors, each with an individual current $i_{m}$ contained in the (M rows x 1 column) vector i. If we define F as a (8 rows x M columns) matrix which describes the strength of each coefficient in the field model generated by a unit current applied to each coil (Rea et al., 2021), then the total amplitude of each component generated by a set of coil currents is

(3) Fi=α.

The coil currents required to cancel (negative sign) the model coefficients α found in Eq. [2] can be obtained by identifying the pseudo inverse matrix $F^{+}$ and evaluating

(4) $$i=-F^{+}\alpha .$$

By combining Eq. [2] and [4], the coil currents which best compensate the uniform field and field gradient components of Δb(t) are given as

(5) $$i=-F^{+}A^{+}\Delta b(t).$$

The quality of the solution (i.e. how well the produced field compares to the target field) will depend on the complexity of the target magnetic field pattern, the geometry of the matrix coil array, the size, shape, and number of unit coils in the array and the proximity of the OPMs to the edge of the volume enclosed by the coil set. The required coil currents may become physically unrealisable as field complexity increases; a constrained solver could be implemented to mitigate this issue, but the produced field pattern may then not be accurate enough for high-quality shielding.

## 3. Methods

### 3.1. Coil design

**Fig. 1.**
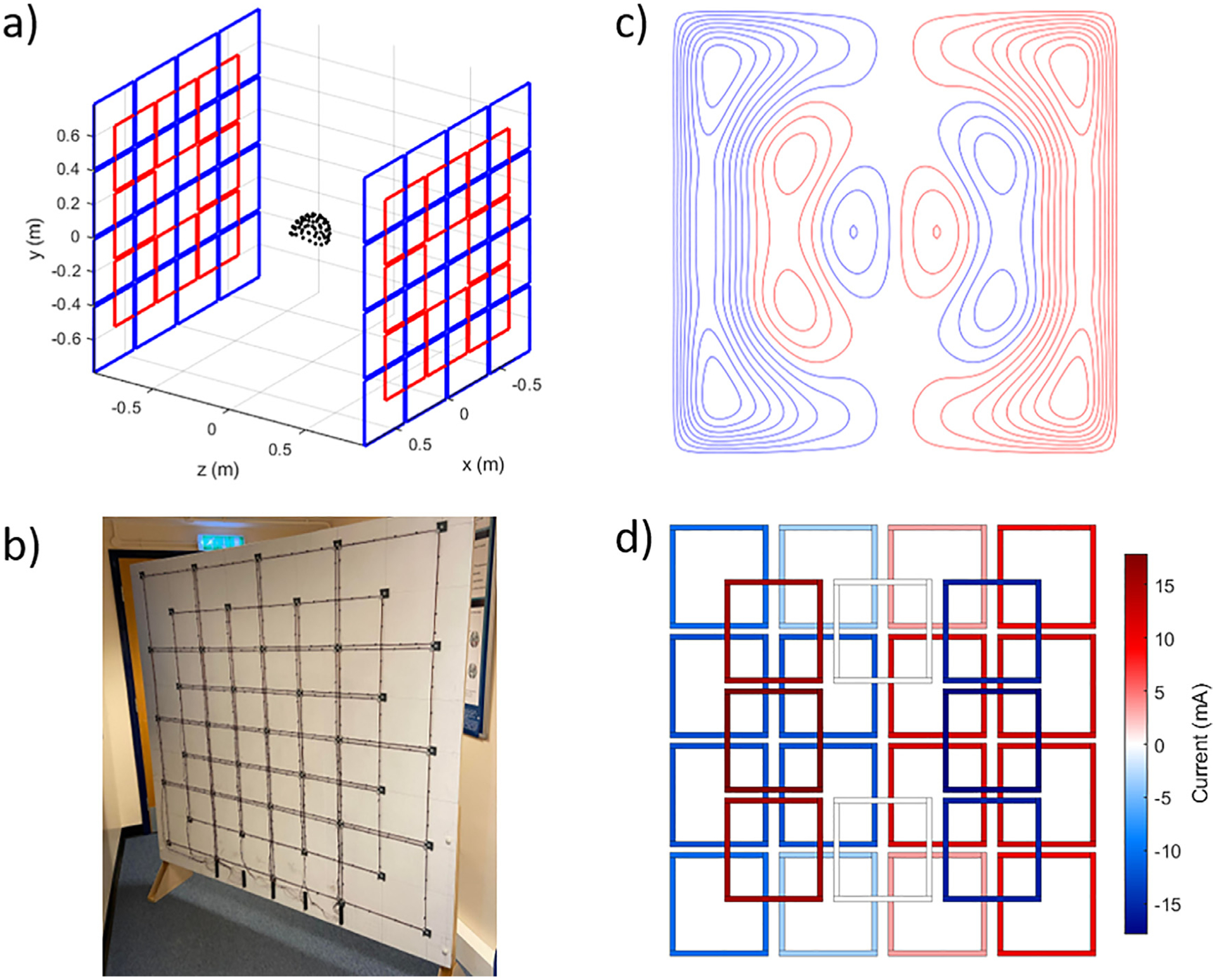
The matrix coil active magnetic shielding system. (a) System is arranged in a bi-planar geometry and each plane contains 24 square coils arranged in an overlapping grid pattern. The blue and red colours highlight the 4×4 grid of coils and the overlapping 3×3 (excluding the central coil) set of coils. The black dots represent the positions of OPM sensors in a 3D printed helmet placed at the centre of the coils. (b) Photograph of a single plane of the constructed coil system. c) the distributed windings of one plane of a bi-planar coil designed to generate a uniform magnetic field in the x-direction (see (a)) over a 40×40×40 cm^3^ volume at the centre of the two planes with deviation <5% from the target magnetic field. Such designs have been used in previous OPM-MEG experiments. Red and blue denote regions of opposing current flow. The second plane (not shown) features the same windings, but the current directions are reversed with respect to the first plane. (d) The currents applied to one plane of the matrix coil to generate the same field as the coil shown in (c), note the similarity between the two designs in terms of current distribution and direction of current flow. The current directions in the second plane are again reversed.

We used a system of 48, 38.5-cm square, coils mounted on two $1.6\times 1.6{\;m}^{2}$ planes separated by 1.7 m as shown in Fig. 1a. Each plane contained 24 coils arranged as a 4×4 grid that filled each plane, along with an overlapping 3×3 grid (excluding the central coil) offset by half the grid spacing. The overlapping grid helps to provide regions of opposing current flow that are required to generate magnetic fields along all three Cartesian axes, despite the coil windings being constrained to a single plane. This design mimics the bi-planar coils described in our previous work (Holmes et al., 2019, 2018), allowing the generation of coil current distributions which approximate the distributed windings as shown in Fig. 1c–d. The system was constructed using two wooden boards, a photograph of one plane of the completed system is shown in Fig. 1b. Plastic plates with wire guides were attached to the corners where each coil was to be mounted to aid winding. Each unit coil was wound by hand, using 10 turns of 0.56 mm diameter enameled copper wire (unit coil resistance ~ 2 Ω, unit coil inductance ~160 *μ*H, coil mutual inductance is maximum between adjacent elements and is <10 *μ*H). Each unit coil was connected to a series of terminal blocks at the base of the large boards by twisted pair windings. When positioned inside the MSR, the terminal blocks were connected to a series of multi-core cables which exited the MSR to be connected to the driving electronics.

### 3.2. Driving electronics

The low-noise coil drivers were constructed in house. The drivers consisted of 12, 4-channel, amplifier boards each operated in voltage feedback mode. Each amplifier channel uses LM324A op-amps (Texas Instruments, Texas, USA) with a 220 Ω resistor added in series to limit current outputs to ±45 mA per channel from ±10 V input voltage (the series resistor reduces magnetic field fluctuations induced by voltage noise from the DACs). Voltage inputs to the drivers were provided by three National Instruments (NI, Texas, USA) NI-9264 16-bit, ±10 V, 16-channel, digital to analogue converter (DAC) cards mounted in a NI-cDAQ-9174 data acquisition (DAQ) chassis interfaced with the NI-DAQmx package in MATLAB (MathWorks Inc., Natick, MA, USA).

### 3.3. OPMs and helmet

An array of QuSpin Inc. OPMs (Zero Field Magnetometer, 3rd generation, triaxial variant) were used for these experiments. The array was housed in a MSR with 4 layers of MuMetal and 1 layer of copper with internal dimensions of $3\times 2.4\times 3{\;m}^{3}$ (MuRoom, Magnetic Shields Limited, Kent, UK) which was demagnetised prior to the start of each experiment leaving a remnant field of ~2 nT at the centre of the room. Each OPM has three, orthogonal, on-sensor coils which cancel the field experienced by the cell via the QuSpin ‘Field Zeroing’ process. The sensors then measure changes in magnetic field relative to this zero point. The field zeroing and calibration of the array was performed using the manufacturer’s software (QuSpin ZFM UI V9) The analogue output of each sensor was connected to a series of NI-9205, 16-bit, 32-channel, analogue to digital converter (ADC) cards mounted in a NI-cDAQ-9179 DAQ chassis. Each channel was sampled at 1200 Hz using the NI-DAQmx package in MATLAB. The sensors were mounted in a rigid, 3D-printed helmet (Cerca Magnetics Limited, Nottingham, UK) such that the position and orientation of each of the three measurement axes of each sensor, were known in the coordinate frame of the helmet model (Hill et al., 2020).

### 3.4. Optical tracking

To calibrate the system via simulation of unit coil fields, we need to know the position and orientation of the OPMs in the helmet with respect to the unit coils. We used an optical tracking system (OptiTrack, Flex13, NaturalPoint Inc., Oregon, USA) which uses a series of six cameras mounted around the MSR to track the position of a series of infrared reflective markers. Such cameras have been used in previous OPM-MEG studies. Aside from a sharp interference peak at the frame rate of the camera (120 Hz) they do not otherwise interfere with the OPM data (Holmes et al., 2018; Roberts et al., 2019; Seymour et al., 2022, 2021). A rigid body of three or more markers (which do not move with respect to each other) can be formed to provide 6-degree-of-freedom (pitch (nodding the head), yaw (shaking the head) and roll (tilting the head) rotations and translations) tracking of the centre of mass of the markers. Here we used a fixed marker set of 5 markers on one coil plane to act as a static reference. A second rigid body was formed by placing 5 markers at known positions on the helmet. We used the NatNet SDK 4.0 software package to interface Motive (the optical tracking camera software, NaturalPoint Inc.) with MATLAB. Specifically, data are streamed from Motive such that MATLAB can poll motion capture data at any time in order to obtain the position of the two rigid bodies within a single frame of the camera. By prior measurement of the coil plane positions with respect to the MSR we applied a coordinate transform to place the helmet and coil rigid bodies into the MSR frame of reference (Rea et al., 2021).

### 3.5. Implementing the nulling

The nulling process was implemented in MATLAB. Firstly, the known position of each sensor with respect to the coil set was obtained by polling the optical tracking data stream and computing the coordinate transform. We then used the Biot-Savart law to calculate the magnetic field generated by a unit current in each unit coil at each sensor. These changes are decomposed into the uniform field and field gradient spherical harmonics (using the centre of mass of the OPM-MEG helmet as the origin of the model) (Mellor et al., 2021; Rea et al., 2021) and are used to form the matrix F (and its corresponding pseudo-inverse matrix $F^{+}$). We then use the helmet positions to form the matrix A (and its corresponding pseudo-inverse matrix $A^{+}$) and calculated Δb as the difference between the mean of the most recent 30 samples of incoming data (30 samples collected at 1,200 Hz sample rate is equivalent to 25 ms of data, an effective data rate of 40 Hz) from each channel and the mean of the first 30 samples of the recording such that the matrix coil is only compensating the change in field experienced by the array relative to its starting field, rather than the absolute remnant field over the helmet. By using the measured resistance (R) of each coil circuit, the required nulling voltages can be calculated as

(6) $$V=Ri=-RF^{+}A^{+}\Delta b.$$

As the process is continuous, and will be sensitive to noise in the data and inaccuracies in the modeling, the voltage at timepoint k is updated with a gain $K_{p}(<1)$ such that

(7) $$V_{k+1}=V_{k}-K_{p}RF^{+}A^{+}\Delta b.$$

**Fig. 2.**
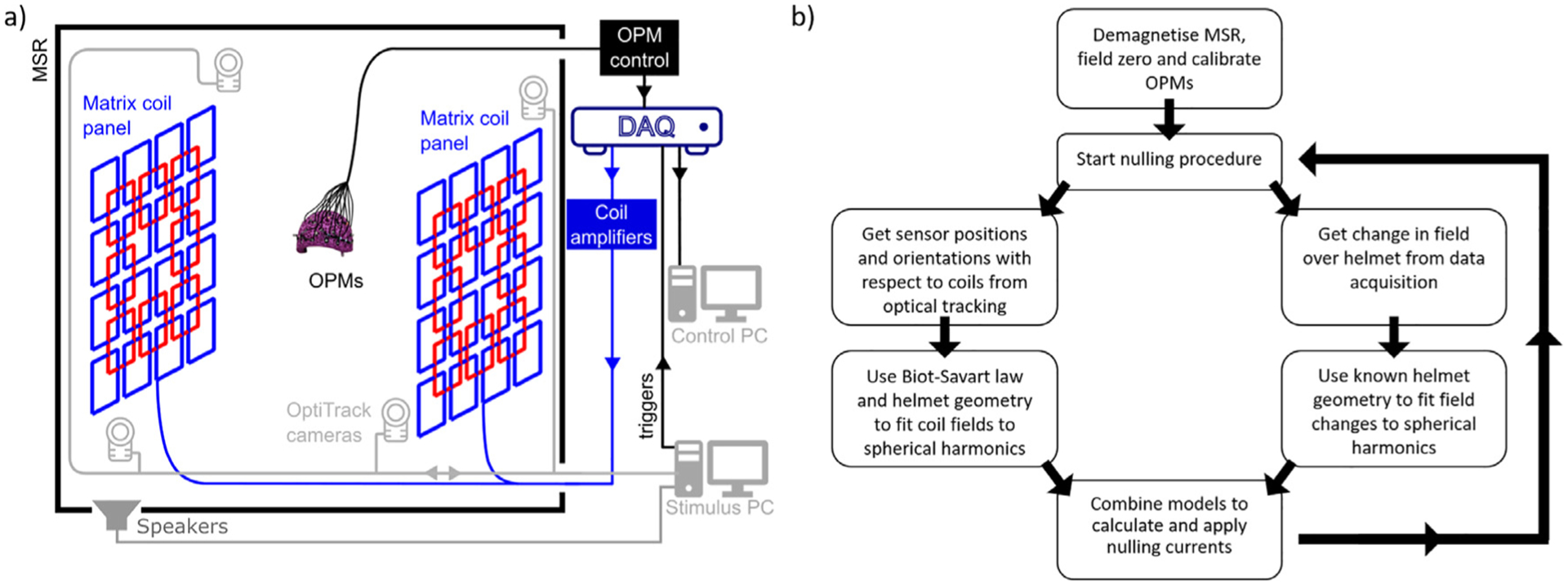
System schematic and nulling procedure flowchart. (a) Overview of OPM-MEG system with matrix coils. The system is housed in a magnetically shielded room. Coil panels are placed either side of a participant wearing a helmet that contains OPMs. A control PC is used to read incoming data from the OPMs and optical tracking cameras then combine these data to calculate and apply nulling currents. A separate PC is used during MEG experiments to provide (in this case) auditory stimulation to a participant via a set of speakers. Trigger channels are used to synchronise the stimuli to the OPM recording. (b) Flowchart of continuous field nulling process.

A limit is placed such that if any value of V exceeds the 10 V range of the DAC it is limited to 10 V. The nulling voltage updates at a rate of 40 Hz, the program stores helmet position information and applied coil currents at the same rate. The rate limiting step is the time taken to calculate the field from the unit coils. Vectorization and pre-calculation of other information was used to focus on this step during nulling. The value of $K_{p}$ was empirically chosen as 0.4. If $K_{p}$ is too large the system will become unstable resulting in a large low-frequency oscillation in the OPM data. This behavior is common in poorly tuned feedback controllers. If $K_{p}$ is too small, then minimal shielding occurs. Fig. 2a shows a schematic overview of the system, and Fig. 2b shows a flowchart of the nulling process.

## 4. Results

### 4.1. Compensating field drifts measured by a stationary array

**Fig. 3.**
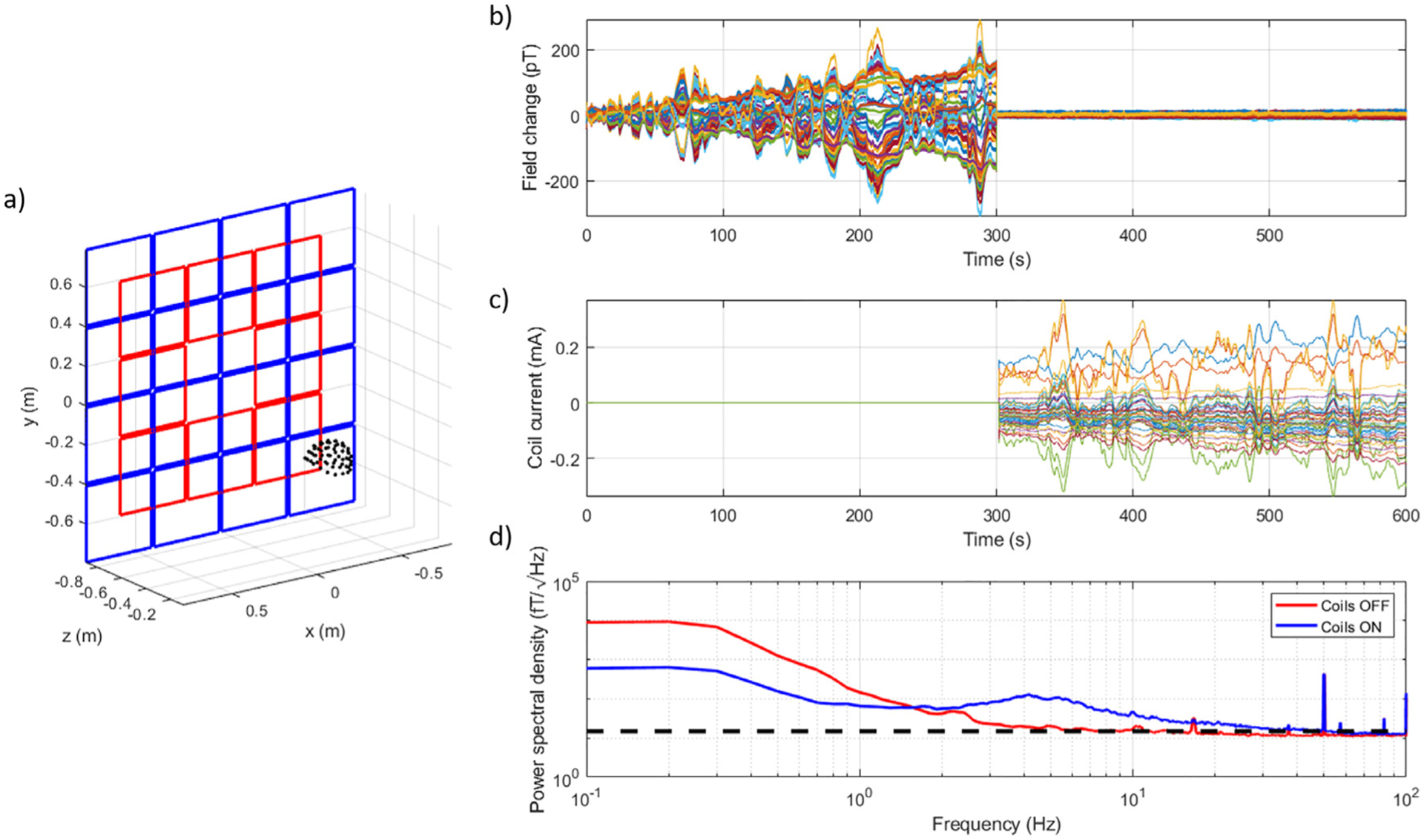
Compensating magnetic field drifts on a fixed array of OPMs. (a) The position of the helmet containing 15 triaxial OPMs with respect to one plane of the matrix coils. The helmet is deliberately positioned off centre in the x, y and z directions to showcase the flexibility of the matrix coil system to generate the required compensation fields at multiple locations between the planes (helmet centre of mass (x,y,z) = (0.15, −0.24, 0.18) m). (b) Magnetic field drifts recorded by the OPMs over a period of 600 s. Each colour represent an individual channel. For the first 300 s the matrix coil system was not active, for the final 300 s the matrix coil compensated changes in the uniform field and field gradients over the helmet. (c) The current applied to each of the 48 coils throughout the experiment. Each colour represents an individual coil current. Note the similarity between the profile of the field changes in the first half of the experiment and the current variations in the second half of the experiment. (d) The power spectral density of the data measured by the OPMs with the coils off (red) and with the coils on (blue). The low-frequency noise level is reduced by the matrix coil, but an increase in noise is seen at higher frequencies. The black dashed line indicates 15 fT/√Hz, the noise floor of the OPMs used in the experiments.

We placed 14 triaxial OPMs into the rigid helmet, giving 42 measurement channels. Sensor positions were evenly distributed to characterise the field and field gradient over the helmet. The helmet was deliberately placed away from the centre of the matrix coil, as shown in Fig. 3a, to demonstrate the system’s flexibility. For all experiments, the MSR was demagnetised, and the OPMs were zeroed and calibrated prior to recording. Data were collected for 600 s. For the first 300 s the matrix coil system was not active (0 mA applied to each unit coil) and for the final 300 s the matrix coil was activated to cancel the changes in field and field gradient over the helmet.

Fig. 3b shows the change in field measured by the OPMs during the experiment. The absolute maximum change in field is reduced from 310 pT to 14 pT, a shielding factor of 13.7. Fig. 3c shows the currents applied to the unit coils throughout the experiment. Nulling is achieved with an absolute maximum current value of 0.37 mA. Note the similarity of the variation of the applied coil currents in the second half of the recording with the field variation manifested in the first half of the recording. Fig. 3d shows the median value of the power spectral density of the data across all sensors for each half of the experiment separately. Significant reduction in the low frequency (0.1 – 1 Hz) fluctuations is achieved with the noise level rising at higher frequency. The broadband interference is a consequence of the changes in magnetic field induced by the matrix coils over the helmet. The strength of this interference is determined by the strength and the rate of change of the artefacts being compensated (as the matrix coil field changes will increase accordingly), and the bit-depth of the DACs (the smallest field variation that can be accurately corrected is set by the field amplitude corresponding to the least-significant bit; this should ideally be smaller than the OPM noise level, but this is not the case here due to the use of 16-bit DACs and the coil drivers’ large dynamic range, which is needed to compensate a wide range of magnetic field strengths during participant movements).

### 4.2. Compensating artefacts on a moving array

The same helmet was then worn by a participant (male, 28, righthanded). The participant was instructed to complete a series of rotations and translations of their head about each axis in turn while seated at the centre of the matrix coil system. Data were recorded for 120 s; for the first 60 s the matrix coil was not active and for the final 60 s the matrix coil was used to compensate changes in the field and field gradient over the helmet. Rotation and translation with respect to each axis was performed twice in each half of the experiment.

**Fig. 4.**
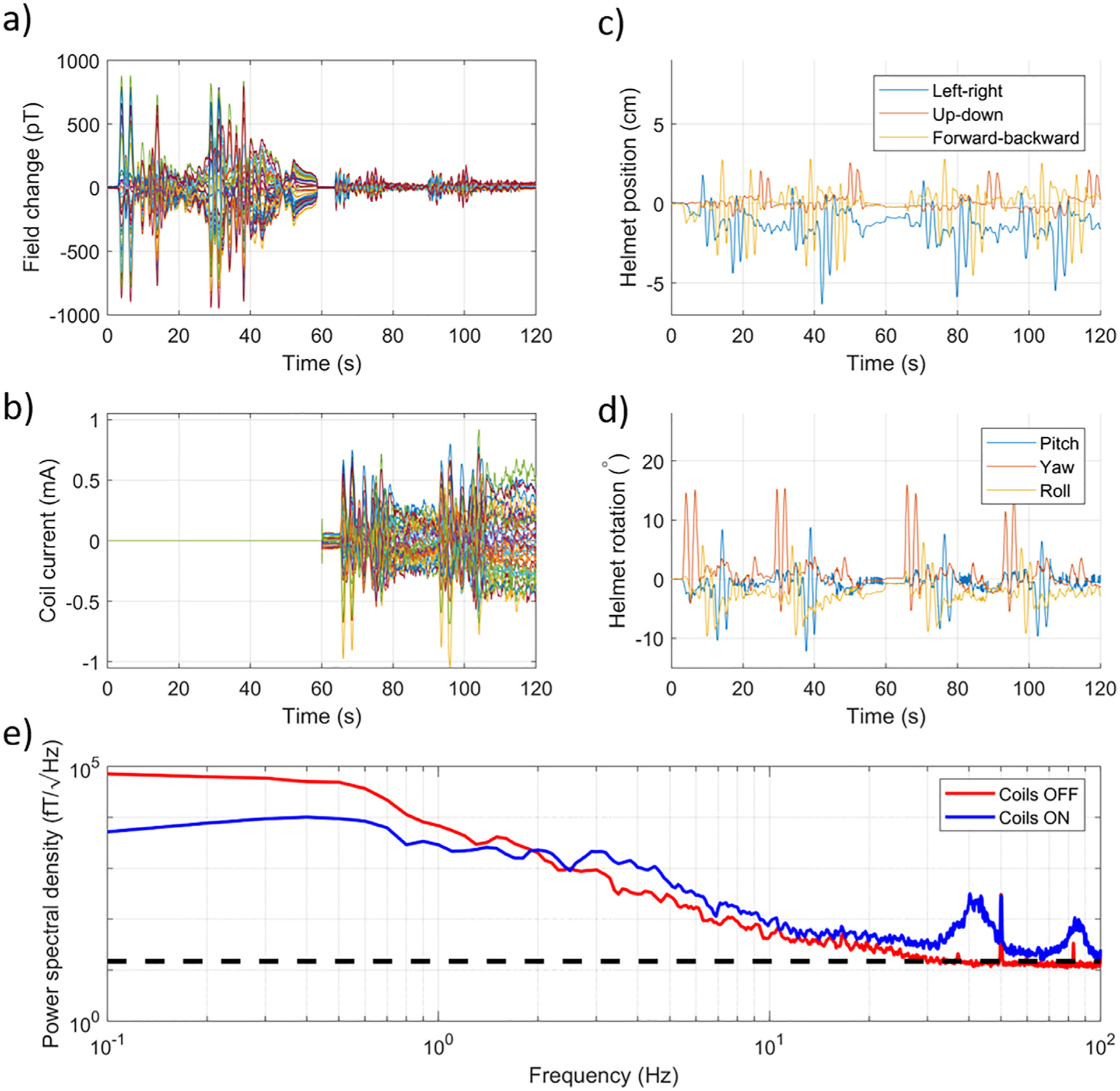
Compensating magnetic field changes on a moving array of OPMs. (a) Magnetic field changes recorded by the OPMs over a period of 120 s whilst a seated volunteer made controlled head movements. For the first 60 s the matrix coil system was not active, for the final 60 s the matrix coil compensated changes in the uniform field and field gradients over the helmet. (b) The current applied to each of the 48 coils throughout the experiment. (c) The change in the position of centre of mass of the helmet during the experiment. (d) The rotation of the helmet about the centre of mass during the experiment. Note the size of the movement remains similar throughout the recording, but the amplitude of the artefact is significantly reduced by the matrix coil in the second half of the recording. (e) The power spectral density of the data measured by the OPMs with the coils on (blue) and with the coils off(red). Compensating a stronger and more quickly varying field results in a large artefact at 40 Hz (the rate at which coil currents are updated). The black dashed line indicates 15 fT/√Hz.

Fig. 4a shows the change in field measured by the 15 OPMs during the experiment. The absolute maximum change in field is increased compared to the static case (Fig. 3b) due to helmet movement and was reduced from 952 pT to 185 pT by the matrix coil system, a shielding factor of 5.2. Fig. 4b shows the currents applied to the unit coils throughout the experiment. Nulling is achieved with an absolute maximum current value of 1.05 mA. Again, note how the variation in the measured fields due to helmet movement is mimicked by the applied coil currents. Fig. 4c shows the change in position of the centre of mass of the helmet during the experiment. Fig. 4d shows the change in rotation of the centre of mass of the helmet during the experiment. Table 1 shows the range of translation and rotation during the experiment. Helmet movements were consistent in the two halves of the experiment, but the measured motion artefact was reduced when the matrix coils were on. Fig. 4e shows separate plots of the median value of the power spectral density across all sensors for each half of the experiment. We note the increase in noise level for both recordings compared to the results shown in Fig. 3 due to the brain noise from the volunteer. Again, the coil system reduces the noise level for low frequency field variations, but the noise is increased at higher frequencies. As the field changes induced in this experiment are stronger, and have a higher rate of change of field, than in the fixed helmet case, the matrix coil generates higher amplitude interference in the measured data, producing a broad ‘bump’ in the noise spectra at 40 Hz, with a harmonic at 80 Hz. The 40 Hz bump appears as the magnetic field produced by the matrix coils is being updated at this rate. This results in discontinuous ‘steps’ of magnetic field in the magnetometer time-course data. These steps have a varying amplitude, but an effective frequency of 40 Hz. As the steps are irregular and not sinusoidal, a broadened peak centred at 40 Hz is produced in the PSD (with a corresponding harmonic at 80 Hz).

### 4.3. Preventing motion-induced sensor gain changes: phantom study

**Fig. 5.**
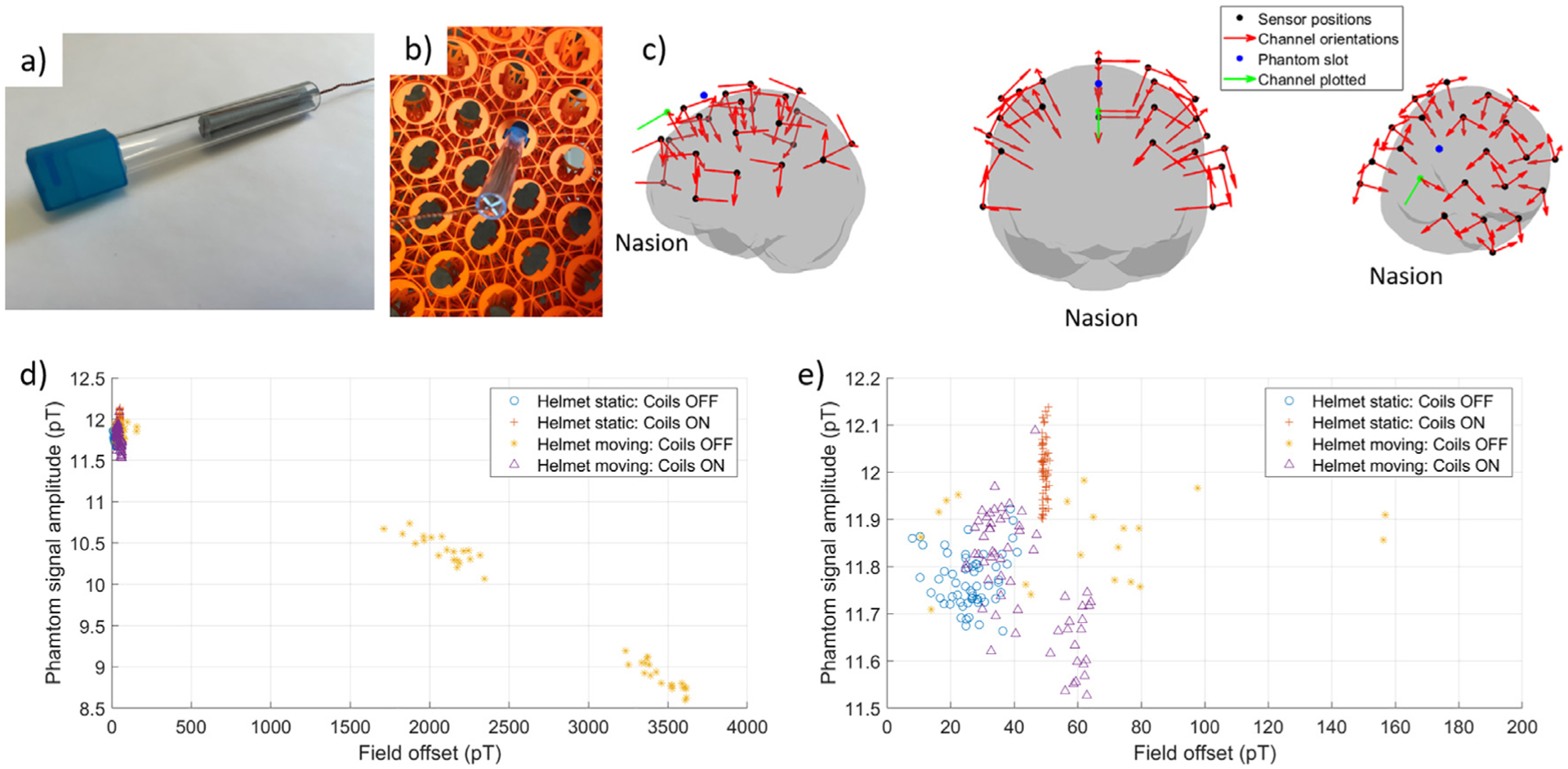
Dry current dipole phantom and sensor gain experiments. (a) Photograph of the dry-type current dipole phantom used in these experiments. The phantom coil is an isosceles triangle wound around a (grey) plastic former with base 5 mm and height 65 mm. An empty (blue) OPM casing is attached to a (clear) Perspex cylinder into which the phantom coil is glued. (b) Photograph of the phantom inside the OPM-MEG helmet. The empty OPM casing can be inserted into any slot in the helmet, the base of the coil is 30 mm beneath the inner surface of the helmet. (c) Each of the 23 triaxial OPMs (black dots) used in the experiment are shown around the template brain, which was used to design the helmet surface. Red arrows indicate the three channel orientations for each OPM. The slot into which the phantom was inserted is shown in blue. (d) Plot of the field amplitude measured on a single channel (indicated by the green dot and arrow in (c)) due to a 200 nA, 23 Hz phantom signal, Results are shown for four experimental cases. Note that during the experiment in which the helmet moved but the matrix coils were not active a large range of field offsets and phantom signal amplitudes are recorded due to sensor gain changes as the field experienced by the OPM moves away from zero. (e) Zoomed in plot of data for low field offsets (0 - 200 pT) to show consistency of measured source amplitude in the other three experiments.

To investigate the capability of our system to collect high-quality MEG data despite significant participant motion, and the presence of broadband artefacts induced by the matrix coil system, we conducted a phantom study. A dry-type current dipole phantom was constructed according to the design used by Oyama et al. (2015). Here, the dry current dipole phantom is an electromagnetic coil with the wirepath forming an isosceles triangle, with 5 mm base and 45 mm height. A 3D-printed plastic former was constructed with slits to guide the winding of a single turn of 0.56 mm diameter enameled copper wire as shown in Fig. 5a. The remaining wire was twisted together to avoid production of stray magnetic fields. To ensure the dipole phantom remained fixed in position with respect to the OPMs in the helmet during movement a Perspex cylinder was connected to an empty OPM casing into which the phantom was glued, the casing could then be fixed into any slot in the OPM-MEG helmet. The base of the current path was positioned 30 mm beneath the inner surface of the OPM helmet and was orientated tangentially to the helmet surface to mimic a neuronal source. The dipole phantom was inserted into a centrally positioned slot within the helmet as shown in Fig. 5b.

We conducted a simple experiment to measure the effects of motion through a non-zero background field on OPM data. The OPM-MEG helmet containing the phantom and 23 triaxial OPMs (69 total channels, with sensor positions and orientations shown in Fig. 5 c relative to the surface of the brain of the template head which was used to design the helmet (see (Hill et al. 2020) for details on how the generic OPM-MEG helmets are designed)) was positioned at the centre of the matrix coil system. The helmet was initially positioned as if a participant was facing forward (Position 1). The MSR was demagnetised and a small (~ 5 nT) magnetic field orientated in the z-direction was applied using a (distributed winding) bi-planar coil (Holmes et al., 2019). The OPMs were field zeroed and calibrated in this $B_{z}$ field at Position 1 prior to the start of the experiment. A 23 Hz sinusoidal current was then passed through the phantom (amplitude 200 nA, producing a maximum measured field of 20 pT). This current was applied for 4.2 s, followed by a 2 s ‘rest’ period during which no current was applied to the phantom. The waveforms were generated by MATLAB interfaced with a NI-9264 DAC at a sample rate of 10 kHz, and a trigger signal was used to synchronise the OPM data with the timings of the current waveform applied to the phantom. An audio cue (also generated by MATLAB) then instructed an experimenter inside the MSR to rotate the helmet (as if shaking the head to the right) by approximately 25° (Position 2). After 5 s had passed the experiment was repeated, and an audio cue instructed the experimenter to rotate the helmet a further 25° (Position 3, ~50° rotation from Position 1). The process was repeated 60 times cycling through each position in the order: 1, 2, 3, 3, 2, 1, 1, 2, 3, 3, etc. such that 20 trials were collected in each position. The experiment was repeated with and without the matrix coils active. For the experiment without the matrix coils, the OPMs were operated in a mode where the operational range is increased from ±1.5 nT to ±5 nT(0.33x gain mode button on QuSpin UI, dynamic range is increased at the expense of bit-precision and effects of sensor gain changes). The increased dynamic range was needed to accommodate the larger field changes without signal clipping. We also conducted two further experiments where all 60 trials were collected whilst the helmet remained fixed in position 1 with, and without, the matrix coils active. We hypothesised that with the matrix coils inactive, movement in the 5nT B_z_-field would produce changes in the measured amplitude of the phantom signal, due to sensor gain changes resulting from variation in the ambient field experienced by the sensors. In contrast, with the matrix coils active, the amplitude of the signal from the phantom was expected to be relatively unaffected by movements. We further hypothesised that these gain changes would affect the accuracy of source reconstructions due to discrepancies between the measured data and the MEG forward model (Borna et al., 2022; Nugent et al., 2022).

#### 4.3.1. Effects of sensor gain changes

From the known timings of the experiment, we first segmented data into trials (each trial was 6.2 s, 0 s - 4.2 s was the sinusoidal waveform with the final 2 s as the rest period) using the trigger signals. For each trial, from each of the four experiments, we then extracted the magnetic field measured by each of the 69 OPM channels during the final 2 s of the 4.2 s ‘stimulation’ period (to isolate a period where the helmet was relatively still to ensure the FFT amplitude was not affected by movement artefacts). For each chunk of data, we calculated the mean value (which we refer to as the field offset, the change in field relative to the initial field zero point of the sensor) and extracted the amplitude of the 23 Hz peak by computing the fast Fourier transform. Fig. 5 d and 5e show the amplitude of the phantom signal as a function of the field offset for the channel (highlighted in green in Fig. 5c) with the largest absolute field offset value in the experiment when the helmet was moving without the matrix coils active. This reflects the worst-case scenario, in fact the total absolute maximum field offsets across all channels were between 325 and 3620 pT, with the values dependant on the orientation of the channel’s sensitive axis with respect to the axis of rotation (~about the vertical y axis) and the applied magnetic field $\left(B_{z}\right.$, meaning channels with an ~y orientation will see little effect whilst sensors with ~X-Z orientations will experience larger field changes). Table 2 summarises the range of motion parameters of the helmet, along with the field offset values and phantom signal amplitudes for the channel shown in Fig. 5c, for all four experiments. We note that despite similarly sized rotations and translations in both movement experiments, in the case where the helmet moves without the matrix coils active the mean and standard deviation of the amplitude of the phantom signal for positions 1, 2 and 3 respectively were 11.9 ± 0.1 pT, 10.4 ± 0.2 pT and 8.9 ± 0.2 pT compared to 11.8 ± 0.1 pT, 11.8 ± 0.1 pT and 11.6 ± 0.1 pT for the experiment repeated with the matrix coils active. The range of field offset values across all positions was decreased from 3605 pT to 39 pT using the matrix coils. The variation in both the mean value and the range of phantom signal strengths are due to sensor gain changes as the background field experienced by the OPM moves away from zero, consequently the true field offset values are likely to be larger than reported. The experiment shows that the matrix coil can compensate the motion-related field changes which induce these gain changes, ensuring the phantom signal is accurately measured.

#### 4.3.2. Effects on source reconstruction

To investigate the effect of the matrix coils and sensor gain changes on reconstruction of the phantom source we separately employed a linearly constrained minimum variance beamformer (Vrba and Robinson, 2001) and a single equivalent current dipole fitting analysis (Hämäläinen et al., 1993).

##### Beamformer analysis.

An estimate of the current dipole strength, ${\overset{ˆ}{Q}}_{\theta }(t)$ is formed at time t for dipole position and orientation θ in a source space (e.g. the brain) using a weighted sum of the measured data as

(8) $${\overset{ˆ}{Q}}_{\theta }(t)=w_{\theta }^{T}m(t)$$

where m(t) is a vector containing the magnetic field measurements recorded by all OPMs at time t and $w_{\theta }$ is a weights vector tuned to θ. The weights are chosen such that

(9) $$\text{min}\left[{\overset{ˆ}{Q}}_{\theta }^{2}\right]\text{s.t.}\;w_{\theta }^{T}L_{\theta }=1$$

where $L_{\theta }$ is the forward field vector containing the solutions to the forward problem for a unit dipole at θ. We note that sensor gain errors affect how well the forward field vector can describe the measured data. The optimal weights vector is expressed as

(10) $$w_{\theta }^{T}={\left[L_{\theta }^{T}C^{-1}L_{\theta }\right]}^{-1}L_{\theta }^{T}C^{-1}$$

where C is the sensor data covariance matrix.

Prior to beamformer analysis we applied homogeneous field correction (Tierney et al., 2021) to the OPM data to remove spatial patterns of field variation over the array that result from uniform magnetic field components (and as such are unlikely to reflect neuronal activity). Homogeneous field correction was also applied to the forward field vectors. Arrays of triaxial OPMs have been shown to have excellent interference rejection properties due to their ability to better distinguish between neuronal sources and external interference (Boto et al., 2022; Brookes et al., 2021; Tierney et al., 2022). We expected the combination of the beamformer, homogeneous field correction and triaxial sensitivity would allow us to recover the underlying MEG signal whilst simultaneously compensating the nulling artefacts.

The source space used here was a regular grid of 4 mm, cubic voxels which span the ‘brain’ of the template head that was used to design the helmet. The forward field vectors were calculated using a single-sphere model and the current dipole approximation (Sarvas, 1987). 1% regularization was applied to the covariance matrices. The source orientation was determined by generalised eigenvalue decomposition as described by Sekihara et al. (Sekihara et al., 2004). Analysis was performed in MATLAB using bespoke software written in house.

We first filtered the OPM dataset between 13 and 30 Hz (using fourth order, Butterworth filtering). Activation maps were then generated by first constructing two covariance matrices for the active and control periods, $C_{a}$ and $C_{c}$ respectively, and then calculating the pseudo-Tstatistical contrast as

(11) $$T_{\theta }=\frac{w_{\theta }^{T}C_{a}w_{\theta }-w_{\theta }^{T}C_{c}w_{\theta }}{2w_{\theta }^{T}C_{c}w_{\theta }}$$

Pseudo-T-statistics were computed at the vertices of the 4 mm grid spanning the entire source space. The active window was chosen to be 2.5 to 3 s following the signal onset and the control window was chosen to be 4.5 to 5 s. The location of the peak activation and corresponding estimated source orientation were then extracted from each activation map.

##### Dipole fitting analysis.

Although the noise rejection properties of the beamformer are likely to be of significant benefit for source reconstructions on low SNR data in the presence of large artefacts, we also implemented a dipole fit to further investigate differences in the source reconstructions due to changes in sensor gain. Specifically, we estimated the position and orientation of a single current dipole with unit (1 nAm) dipole moment that produces the maximum correlation between the lead fields of the simulated source and the measured data. The dipole fitting was implemented using MATLABs constrained minimization function *fmincon*, with constraints on the dipole position such that it could only exist within the source space. For a given source position, the source orientation was estimated by assuming the dipole to be orientated tangentially to the template brain surface and computing a lead field ‘sweep’ for all possible dipole orientations in this tangential plane (between 0 and 180°, in steps of 1°). We then identified the orientation with the highest correlation between the lead field and the measured data and used this value in our objective function. Once the best fitting dipole was found, the dipole moment was estimated via linear regression of the lead field pattern produced by the estimated dipole with the measured data.

##### Quantifying changes.

**Fig. 6.**
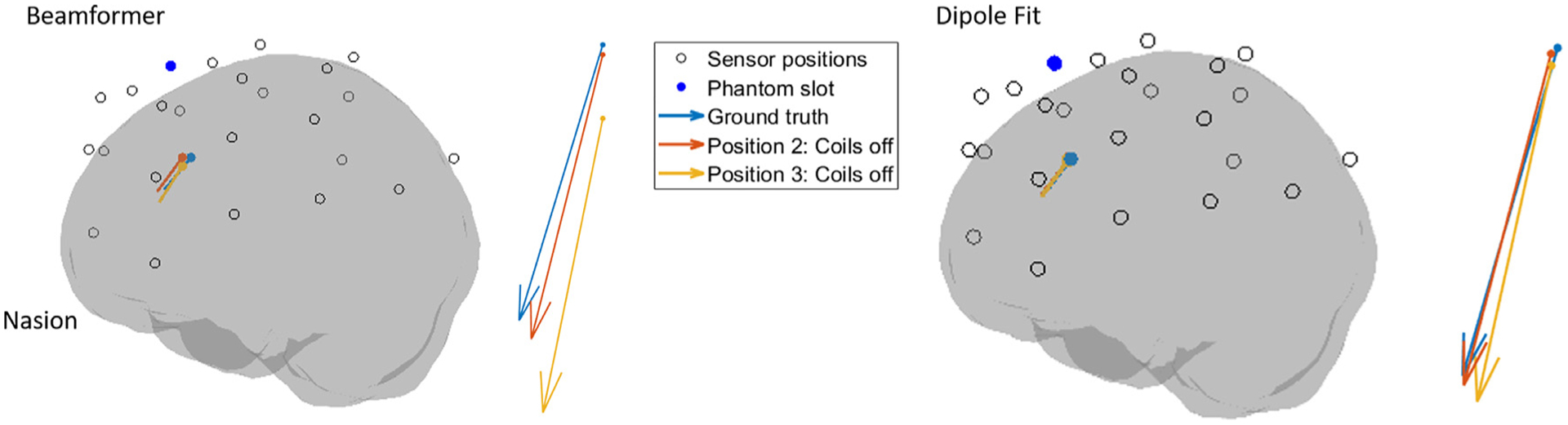
Dry phantom source reconstruction results. The reconstructed source position and orientations are shown for the dipole fitting and beamformer analysis for three cases, black circles show OPM positions and the blue dot highlights the slot into which the phantom was inserted. The first case is when the helmet was static and the matrix coils were switched off, referred to here as the ground truth. In the beamformer analysis, this voxel and approximate source orientation were consistent with the ground truth for all experiments other than trials in positions 2 and 3 during the experiment where the helmet was moving without the matrix coils active. Inset arrows show zoomed in source positions and orientations for comparison between conditions.

For both analyses, we first found the source location and orientation for the experiment in which the helmet did not move, and the matrix coils were not active. These serve as our ‘ground truth’ measurements (note we did not use the measured position of our source in the helmet to compare to as we wished to focus on the difference between datasets) to which all subsequent data are compared. The ground truth data were comparable for beamformer and dipole fitting, source positions: voxel position [3.50,19.5,42.5] mm for the beamformer and position [3.32,21.5,42.4] mm for the dipole fit, with source orientations: [0.26,0.58,–0.77] for the beamformer and [0.25,0.56,–0.79] for the dipole fit, the estimated dipole moment was 1324 nAm. Table 3 summarises the Euclidean distance between the peak location (peak voxel location for beamformer analysis) and the ground truth values, the angle between the orientation vectors and the ground truth values and the estimated dipole moments for all conditions and experiments. The results show that for the experiment in which the helmet remained still, but the matrix coil was active, the beamformer was able to localise the phantom to the same 4 mm voxel as found in the ground truth measurement, with a change in orientation of just 0.05°, compared to a change of 0.067 mm and 0.046° and no change in dipole moment for the dipole fit. For the moving phantom experiment during which the matrix coil was active, the source was again localised to the same voxel as the ground truth for all positions with a change in orientation of 0.29°/0.93°/1.50° for Positions 1/2/3 respectively, compared to a change of 0.16/0.27/0.49 mm and 0.12°/0.95°/1.85° for the dipole fit. The dipole moments were 1325/1326/1327 nAm for each position showing consistency with the ground truth. For the moving phantom experiment without matrix coils active, the source reconstructed from data collected in Position 1 (maximum field offsets for channel shown in Fig. 5c were <160 pT) localised to the same voxel as the ground truth with a change in orientation of 0.04°, compared to a change of 0.27 mm and 0.17° for the dipole fit. The source reconstructed from data collected in Position 2 (maximum field offsets were between 1700 and 2350 pT) was 4 mm away from the ground truth with a change in orientation of 3.16°, compared to a change of 0.95 mm and 1.37° for the dipole fit. The source reconstructed from data collected in Position 3 (maximum field offsets were between 3230 and 3620 pT) was 5.7 mm away from the ground truth with a change in orientation of 7.06°, compared to a change of 1.5 mm and 3.45° for the dipole fit. The estimated dipole moments for each position were 1321/1289/1260 nAm. Fig. 6 shows the difference between the ground truth measurement and the sources reconstructed from data in Positions 2 and 3 during the experiment in which the matrix coil was off. Results show greater changes in source orientation, position and dipole moment from the ground truth for the experiment in which the matrix coils were not active, but the helmet was moving, highlighting the benefits of compensating field changes to ensure accurate source reconstructions.

### 4.4. MEG demonstration

To further demonstrate the capability of the matrix coil system we performed a MEG recording on a participant undergoing ambulatory motion. An array of 33 triaxial OPMs (99 channels) were arranged in the helmet to cover left and right sensorimotor regions. The participant (same participant as in Section 4.2) stood at the centre of the coil system wearing the helmet and holding two button boxes. After hearing an auditory cue indicating either ‘left’ or ‘right’, the participant continually pressed a button using either their left or right index finger until a second auditory cue (after 2 s) instructed the participant to ‘stop’. We expected this experiment to induce a movement related desynchronization and post-movement rebound in the beta band (13–30 Hz) in the contralateral sensorimotor cortex. The process was repeated 60 times, including 30 trials for each hand. Left/right trials were presented in a random order. During the experiment the participant was unconstrained and instructed to walk (exploring only the volume between the coils). The process was repeated with and without the matrix coil system active. The experiment was approved by the University of Nottingham Medical School Research Ethics Committee, the volunteer gave informed consent. For the experiment without the matrix coils, the OPMs were again operated in their 0.33x gain mode.

#### 4.4.1. Sensor level and movement analysis

**Fig. 7.**
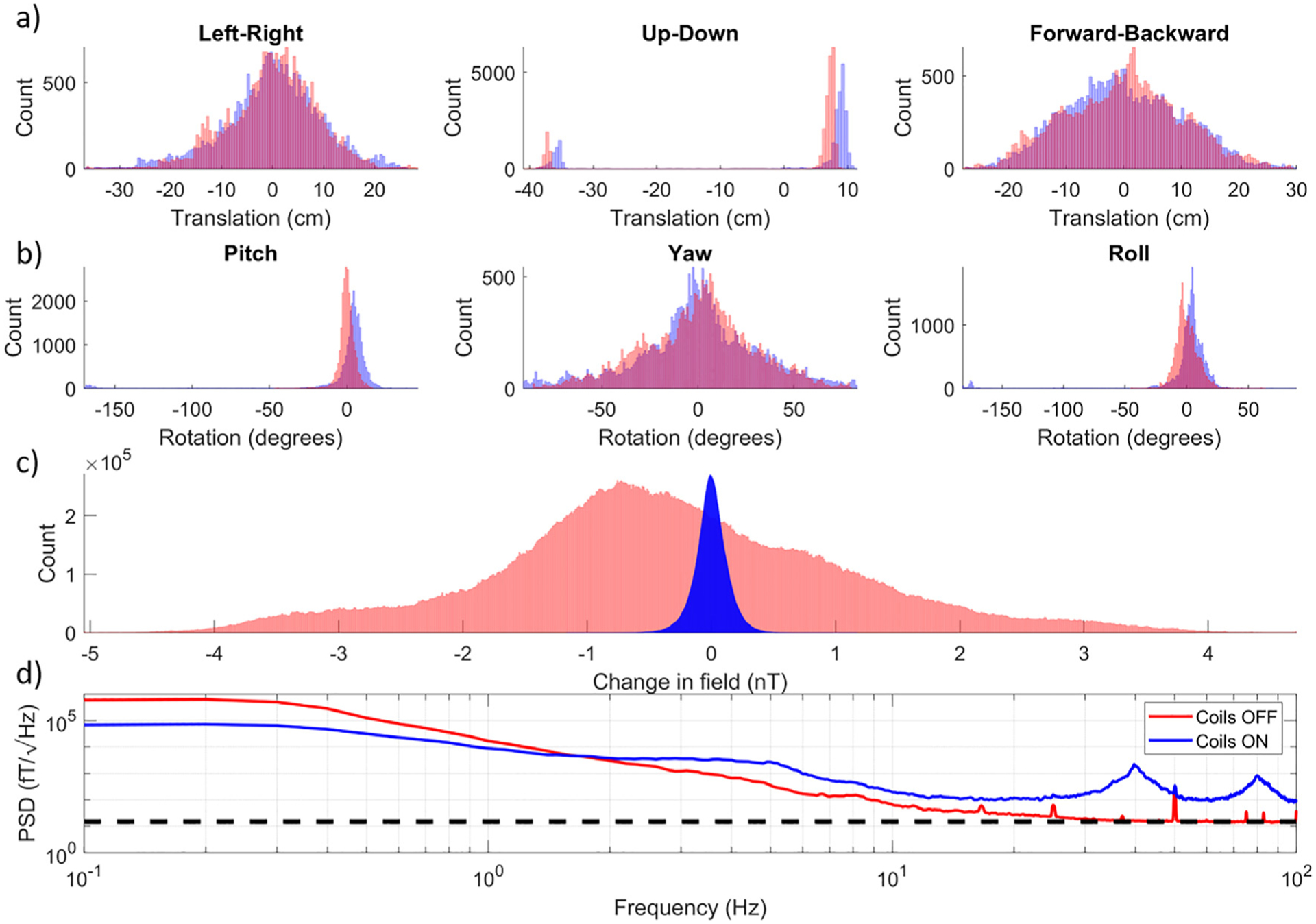
Range of motion and magnetic field change recorded from a participant undergoing ambulatory movements. (a) Histograms of the change in position of the centre of mass of the OPM-MEG helmet relative to its mean position. The three histograms show left-right, up-down and forward-backward movement, respectively. For all plots, blue and red denote the experiments with and without the matrix coils active. (b) Histograms of the change in orientation of the centre of mass of the OPM-MEG helmet relative to its mean orientation. The three histograms show pitch, yaw and roll, respectively. We note that only a short amount of time was spent at the large pitch and roll orientations, and there is a correspondingly low count rate at the extremes of the corresponding histogram x-axis. (c) Histogram of the change in magnetic field measured by each channel of the OPM array relative to the value at the start of the recording. This shows that the matrix coil significantly decreases field variations which are induced by sensor movement. (d) The power spectral density of the data measured by the OPMs in each recording. Compensating large field changes induced by participant movement results in a further increase in both the broadband noise and the 40/80 Hz coil artefacts. The black dashed line indicates 15 fT/√Hz.

Fig. 7a shows histograms of the translations of the centre of mass of the helmet recorded by the optical tracking system with (blue bars) and without (red bars) the matrix coil active. Fig. 7b shows histograms of the rotation about the centre of mass of the helmet recorded by the optical tracking system during the two experiments. In the coils-on condition the participant turned their back relative to their starting position resulting in a lager range of rotation values, this movement was not attempted during the coils-off condition as it would have produced field changes that exceed the maximum measurable field of the OPMs. Fig. 7c shows histograms of magnetometer data collected in the two conditions. The absolute maximum change in field is reduced from >5.00 nT (saturation of some OPM sensors occurred during the recording) to 1.17 nT using the matrix coil system. Despite a similar range of movement, the matrix coil reduces the size of artefacts in the data, ensuring the sensors stay within their operational range and that gain changes are minimised.

#### 4.4.2. Source reconstruction

We used the same beamformer analysis detailed in the previous section for this data. Here, co-registration of the sensor positions to an anatomical MRI of the participant was achieved using optical methods (Hill et al., 2020; Zetter et al., 2019). No regularization was applied to the covariance matrices. The significant movement artefact and coil artefacts made ‘bad trial’ identification challenging, but one trial was rejected from the coils-off dataset where some sensors appeared to briefly malfunction.

**Fig. 8.**
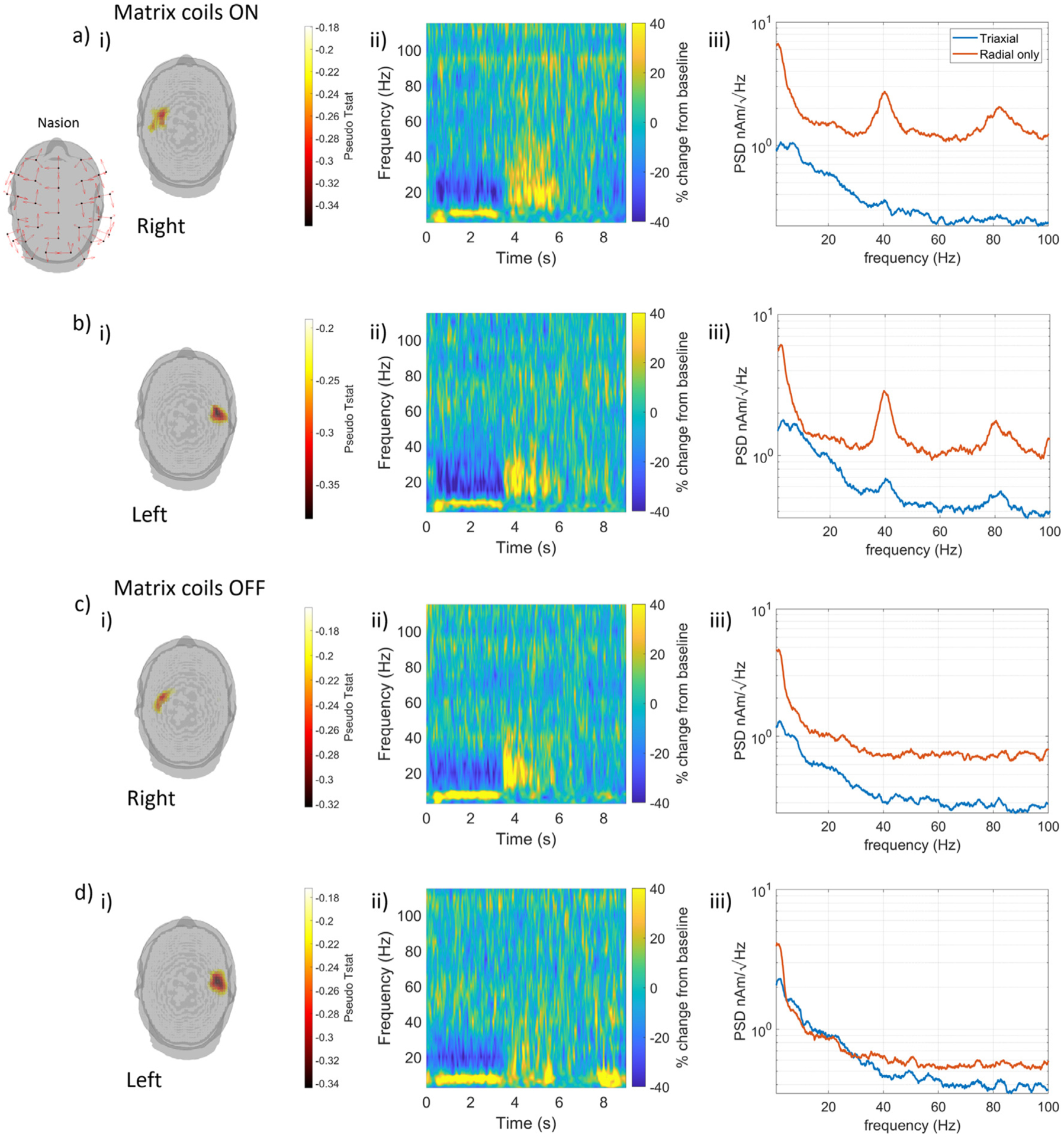
Source reconstruction of MEG data collected from a participant undergoing ambulatory movements with and without matrix coils active. (a) Data from trials in which the participant was pressing the button with their right index finger with the matrix coil active. (b) Data from trials in which the participant was pressing the button with their left index finger with the matrix coil active. (c) and (d) show data from right and left-handed trials during the experiments where the matrix coil was off. For each case: (i) Beamformer images of the spatial signature of beta band modulation (thresholded to 80% of the maximum value) shows activity in the contralateral sensorimotor cortices as expected (channel positions (black dots) and orientations (red arrows) shown inset to (a)). ii) Time-frequency spectra of a virtual electrode at the peak of the beamformer image showing movement related desynchronization (blue) in the beta band and post movement beta rebound (yellow). (iii) Power spectral density of the virtual electrodes reconstructed using a triaxial beamformer and a radial-only beamformer. Note how the triaxial beamformer significantly reduces the impact of the 40 and 80 Hz artefacts generated by the matrix coil.

To generate an image of the spatial form of neuronal activity in the beta band during the experiment, we first filtered the OPM dataset between 13 and 30 Hz (using fourth order, Butterworth filtering). Images of activation that show the pseudo-T-statistical contrast between activity in active and control windows were produced for left and right trials separately. Pseudo-T-statistics were computed at the vertices of a regular 4 mm grid spanning the whole brain of the participant. The active window was chosen to be 1 to 3 s following the start cue (i.e. during the button pressing) and the control window was chosen to be 3 to 4 s (contrasting the desync and rebound periods to maximise the pseudo T value). The grid of T values was thresholded to 80% of the peak value and overlaid onto the anatomical MRI of the participant. For Left (Fig. 8a) and Right (Fig. 8b) conditions with the matrix coils active and the corresponding Left (Fig. 8c) and Right (Fig. 8d) conditions without the matrix coil, Fig. 8(i) shows the activation images from the experiment performed with the matrix coils active. The activity peaks in the contralateral motor regions during the button pressing as expected. Similar activation images are obtained with and without the nulling, though we note that the spread of measured fields shown in Fig. 7 is sufficient to induce sensor gain changes in some data.

The signal at the peak location of each image was reconstructed to form a virtual electrode timecourse, with beamformer weights now calculated in the broad-band by filtering data between 1 and 150 Hz. This virtual electrode was used to generate a time-frequency spectrum (TFS) by filtering the timecourse sequentially into overlapping frequency bands. For each band, the Hilbert envelope was calculated before averaging over trials and concatenating in the frequency domain. A control window of 7 to 8 s after the cue to commence button pressing was used to show change in activity relative to baseline. Fig. 8(ii) shows the TFS for the left and right conditions displaying the expected desynchronization and rebound in beta activity. Finally, virtual electrodes calculated using the full triaxial dataset of 99 channels and separately by using only the component of magnetic field which is radial to the surface of the head (33 channels) were compared to show the effectiveness of the triaxial beamformer in compensating artefacts. The power spectral density of the virtual electrode (across all trials) was computed and plotted for the two conditions in Fig. 8(iii). As expected, the triaxial array is better able to differentiate the broadband noise and the 40 Hz artefact (and its 80 Hz harmonic) from neuronal sources compared to only the radial field. This is because the full vector field measurements enable more information to be gained on the spatial signature of interference (Brookes et al., 2021; Tierney et al., 2022; Rea et al., 2022)

## 5. Discussion

The matrix coil is a flexible, reconfigurable, active magnetic shielding system with application to fixed and moving arrays of magnetic field sensors. The shielding framework outlined here can be readily adapted to any coil system. Application to OPM-MEG in particular shows that high-quality source data can be collected from participants undergoing ambulatory motion. Unconstrained movement during a scan opens a wealth of possibilities for clinical investigation and allows a fundamentally new range of neuroscientific experiments. The framework adopted here could also be expanded to simultaneously null magnetic fields over multiple helmets, facilitating MEG studies of social interaction (KingCasas et al., 2005; Leong et al., 2017; Montague et al., 2002).

Although data could be recorded during the MEG experiment conducted with the coils switched off the effect of non-linearities induced into the sensor must be considered when evaluating the benefits of field nulling using a coil system (Boto et al., 2018; Iivanainen et al., 2019). Results shown here (Figs. 5 and 6) and in other studies suggest these changes negatively impact the quality of source localization, reducing the accuracy of spatial localization of the source position and removing a key advantage of MEG (Nugent et al., 2022). Borna et al. (2022) showed that accounting for non-zero orthogonal field terms induces cross-axis projection error (CAPE) which further degrades the quality of sensor calibration. To minimise CAPE, the authors suggested that field changes should be restricted to ± 1 nT (as was effectively achieved here). Closedloop operation of OPMs (where fields are continually applied to the internal coils) is also affected by CAPE if it is not applied to all three axes. Precision control of the magnetic field environment is therefore crucial for OPM-MEG.

However, several technical issues need to be addressed to fully realise the potential of the matrix coil system if it is to become a standard technique. The available nulling volume must be expanded to allow for a greater range of motion; this could readily be achieved by mounting coils on the walls of the MSR (Holmes et al., 2022) and applying the framework established here. Although they can be corrected with post-processing and source reconstruction, the matrix coils produce both an increase in broadband noise above 1 Hz, and large drive frequency artefacts at 40 and 80 Hz which effects sensor level data. An increased nulling rate would not only improve system performance to address more rapidly varying fields but could also potentially shift the artefact outside the OPM bandwidth (via multiple updates at the same helmet position). Integration with improved hardware such as FPGA controllers should alleviate the problem. Narrowing the artefact peak and shifting it (e.g. such that it overlaps into the powerline artefact or is >100 Hz) may also help minimise its impact. Cancellation of higher order field variations should also be considered; though the magnitude of the required coil currents will increase so coil driver specification and the impact on noise levels in the MSR must be evaluated.

The matrix coils, the method of production of spherical harmonic fields and the shielding framework adopted here could also have application outside MEG. For example, in designing excitation fields for large magnetic induction tomography systems (Cubero et al., 2020; Kim et al., 2019); adaptive nulling for atom interferometers for gravity sensing (Hobson et al., 2022); reducing systematic errors as mobile atom interferometers are deployed in real-world environments (Wu et al., 2019) and for fundamental physics experiments including very long baseline atom interferometry (Wodey et al., 2020) and measurements of the electric dipole moment of the neutron (Afach et al., 2014).

Overall, the matrix coil goes a long way to achieving one of the key benefits of OPM-MEG: unconstrained movement during a scan. As systems continue to develop, and the range of allowed experiments continues to expand, OPM-MEG has significant potential to become the method of choice for functional neuroimaging.

## Figures and Tables

**Table 1:** The range of translation and rotation of the OPM-MEG helmet during the experiments.

	Translations (cm)			Rotations (°)		
	Left-right	Forward-backward	Up-down	Pitch	Yaw	Roll
*Seated head movement*						
Matrix coil ON	8.1	7.0	3.4	21.0	20.9	15.4
Matrix coil OFF	7.3	7.3	3.1	17.3	21.4	15.5
*Ambulatory MEG task*						
Matrix coil ON	57.0	50.5	62.7	215.1	173.4	270.3
Matrix coil OFF	56.7	49.9	64.6	63.00	164.9	108.5

**Table 2:** The range of translation and rotation of the OPM-MEG helmet and the measured amplitude of the phantom signal, and level of field offset recorded during the phantom experiments.

Condition	Translations (cm)	Rotations (°)	Helmet Position	Phantom signal amplitude (pT)	Field offsets (pT)
Static: Coils OFF	n/a	n/a	1	11.8 ± 0.1	26 ± 8
Static: Coils ON	n/a	n/a	1	12.0 ± 0.1	50 ± 1
Moving: Coils OFF	Left-right: 3.4	Pitch: 5.9	1	11.9 ± 0.1	64 ± 41
	Up-down: 0.3	Yaw: 54.0	2	10.4 ± 0.2	2086 ± 170
	Forward-backward: 10.1	Roll: 7.4	3	8.9 ± 0.2	3456 ± 126
Moving: Coils ON	Left-right: 4.6	Pitch: 5.2	1	11.8 ± 0.1	31 ± 3
	Up-down: 0.4	Yaw: 44.1	2	11.8 ± 0.1	39 ± 5
	Forward-backward: 8.9	Roll: 6.0	3	11.6 ± 0.1	60 ± 3

**Table 3:** Effects of helmet movement through non-zero background field on phantom source reconstruction accuracy.

	Beamformer		Dipole Fit		
	Distance from ground truth to estimated source (mm)	Angle between ground truth and estimated source (°)	Distance from ground truth to estimated source (mm)	Angle between ground truth and estimated source (°)	Estimated Dipole moment (nAm)
Ground Truth Recording	n/a	n/a	n/a	n/a	1324
Static: Coils ON	0 (Same voxel)	0.05	0.067	0.046	1324
Moving: Coils OFF Position 1	0 (Same voxel)	0.04	0.27	0.17	1321
Moving: Coils OFF Position 2	4 (1 voxel)	3.16	0.95	1.37	1289
Moving: Coils OFF Position 3	5.7 (2 voxels)	7.06	1.5	3.45	1260
Moving: Coils ON Position 1	0 (Same voxel)	0.29	0.16	0.12	1325
Moving: Coils ON Position 2	0 (Same voxel)	0.93	0.27	0.95	1326
Moving: Coils ON Position 3	0 (Same voxel)	1.54	0.49	1.85	1327

## Data Availability

Data will be made available on request.
